# Induction of T cell exhaustion by JAK1/3 inhibition in the treatment of alopecia areata

**DOI:** 10.3389/fimmu.2022.955038

**Published:** 2022-09-20

**Authors:** Zhenpeng Dai, Tanya Sezin, Yuqian Chang, Eunice Y. Lee, Eddy Hsi Chun Wang, Angela M. Christiano

**Affiliations:** ^1^ Department of Dermatology, Vagelos College of Physicians and Surgeons, Columbia University, New York, NY, United States; ^2^ Department of Genetics and Development, Vagelos College of Physicians and Surgeons, Columbia University, New York, NY, United States

**Keywords:** alopecia aerata (AA), T cell, gamma chain cytokines, JAK - STAT signaling pathway, T cell exhaustion

## Abstract

Alopecia areata (AA) is an autoimmune disease caused by T cell-mediated destruction of the hair follicle (HF). Therefore, approaches that effectively disrupt pathogenic T cell responses are predicted to have therapeutic benefit for AA treatment. T cells rely on the duality of T cell receptor (TCR) and gamma chain (γc) cytokine signaling for their development, activation, and peripheral homeostasis. Ifidancitinib is a potent and selective next-generation JAK1/3 inhibitor predicted to disrupt γc cytokine signaling. We found that Ifidancitinib robustly induced hair regrowth in AA-affected C3H/HeJ mice when fed with Ifidancitinib in chow diets. Skin taken from Ifidancitinib-treated mice showed significantly decreased AA-associated inflammation. CD44^+^CD62L^-^ CD8^+^ T effector/memory cells, which are associated with the pathogenesis of AA, were significantly decreased in the peripheral lymphoid organs in Ifidancitinib-treated mice. We observed high expression of co-inhibitory receptors PD-1 on effector/memory CD8^+^ T cells, together with decreased IFN-γ production in Ifidancitinib-treated mice. Furthermore, we found that γc cytokines regulated T cell exhaustion. Taken together, our data indicate that selective induction of T cell exhaustion using a JAK inhibitor may offer a mechanistic explanation for the success of this treatment strategy in the reversal of autoimmune diseases such as AA.

## Introduction

Alopecia areata (AA) is an autoimmune disease of the hair follicle (HF) which results in hair loss that ranges in presentation from circular patches on the scalp to total scalp hair loss to full-body hair loss (1–4). The etiology of AA has been shown to involve a combination of genetic predisposition and environmental triggers (2–4). Due to the significant psychosocial burden of AA, affected patients experience enormous psychological and emotional stress (5, 6).

We previously showed that cytotoxic NKG2D^+^CD8^+^ T lymphocytes are necessary and sufficient for AA development (7). The pathogenesis of AA is also associated with the overexpression of cytokines, including interferon gamma (IFN-γ) and the common gamma chain (γc) cytokines interleukin (IL)-2, IL-7, and IL-15, which promote the activity of alopecic T lymphocytes in affected skin (3, 4, 7). The IFN-γ and γc cytokines signal through receptors that utilize Janus kinases (JAKs) as their downstream effectors. These signals are subsequently propagated by signal transducers and activators of transcription (STATs) to regulate the expression of associated genes (8). Since JAK-STAT pathways play an essential role in both innate and adaptive immunity, dysregulation of JAK-STAT signaling has been implicated in multiple autoimmune disorders (8, 9). Indeed, inhibition of JAK activity by small molecule inhibitors has demonstrated clinical efficacy in rheumatoid arthritis (RA) and other autoimmune diseases (8–11). Recent progress in treating AA has been demonstrated with JAK inhibitors such as ruxolitinib (a JAK1/2 inhibitor) and tofacitinib (a pan-JAK inhibitor), which both robustly restored hair regrowth in the C3H/HeJ mouse model and in patients with AA (7, 12–14).

Signaling through the six γc cytokines (IL-2, IL-4, IL-7, IL-9, IL-15 and IL-21) requires JAK3 binding to the γc present in the receptor complexes for all six cytokines, as well as JAK1 binding to the other chains within the receptors (5). Through these JAK-receptor interactions, each γc cytokine induces differential activation of STAT pathways to achieve specific effects. Dual JAK1 and JAK3 inhibition can effectively suppress γc cytokine signaling by inhibiting downstream signal transduction (15, 16). Therefore, a selective JAK1/3 inhibitor could provide increased therapeutic efficacy through suppression of AA-associated cytokines (γc cytokines and IFNs) while avoiding unwanted JAK2-mediated side effects (8, 9).

To test this hypothesis, we used Ifidancitinib, a selective next-generation JAK1/3 inhibitor, in the C3H/HeJ mouse model of AA (17). We found that Ifidancitinib inhibited JAK1/3 function *in vitro* and reversed AA in C3H/HeJ mice *in vivo* with reduced pathogenic T cell responses and decreased infiltration of immune cells into the skin. Moreover, the effector/memory T cells exhibited a profile of markers of T cell exhaustion after treatment with Ifidancitinib. We found that blockade of γc cytokine signaling through other JAK1 or JAK3 inhibitors promoted T cell exhaustion. Our results provide rationale for pursuing clinical studies of selective next generation JAK inhibitors to inhibit JAK1 or JAK3 signaling in therapies for patients with AA.

## Results

### Ifidancitinib inhibited JAK1/3 signaling pathways

**Figure 1:**
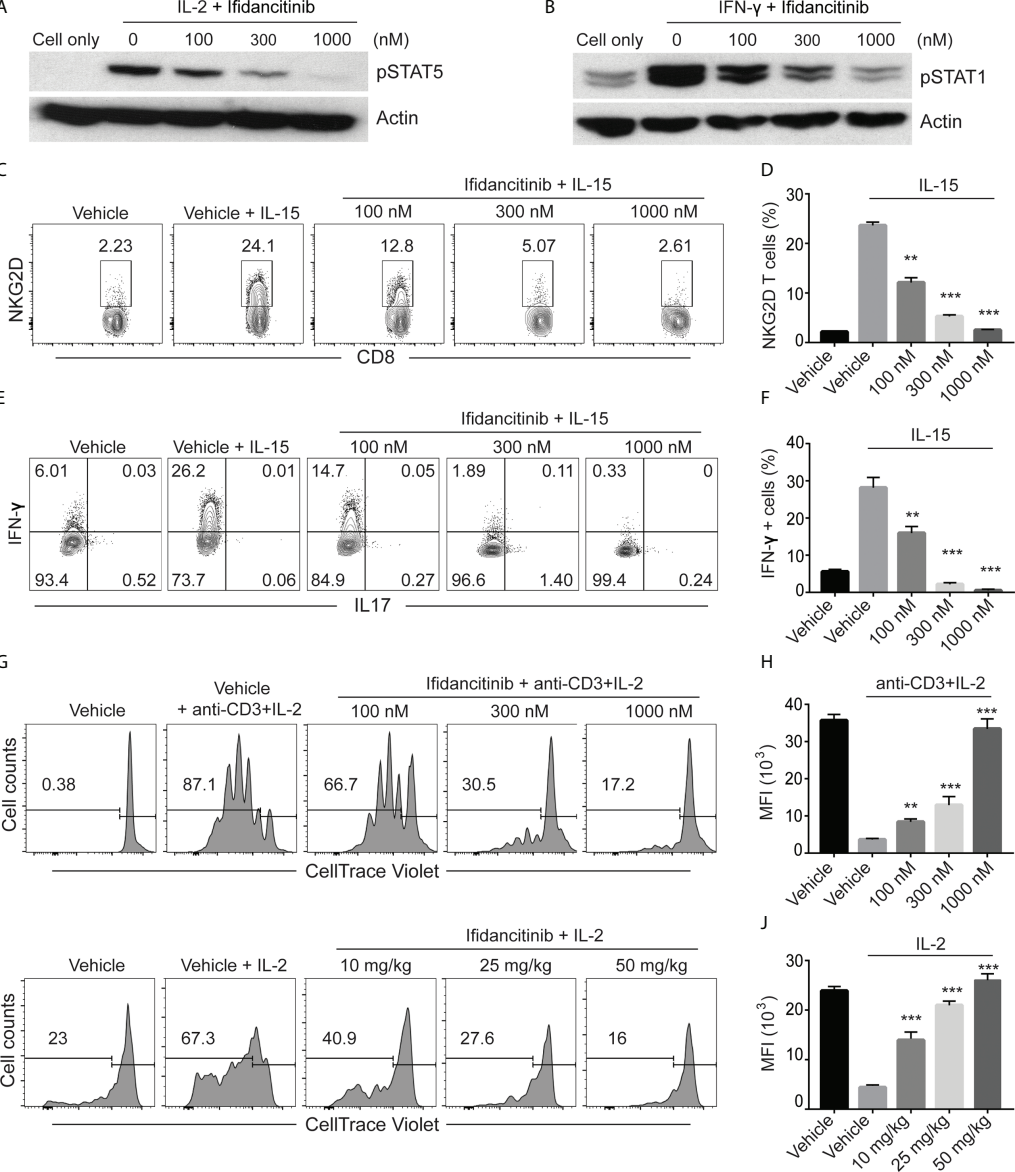
Ifidancitinib inhibited γc cytokine signaling. **(A)** and **(B)** *In vitro* activated T cells were pretreated with various dose of Ifidancitinib or vehicle (DMSO) for 1 h and stimulated with rhIL-2 (20 ng/ml) or rmIFN-γ (40 ng/ml) for 15 min, and then cell lysates were subjected to immunoblotting with the indicated Abs. **(C)** to **(F)** CD8 T cells from normal haired C3H/HeJ mice were stimulated with rmIL-15 (50 ng/ml) in the presence of increasing dose of Ifidancitinib or DMSO for 4 (d) On day 5, NKG2D expression was analyzed by flow cytometry **(C)** and **(D)**. In **(E)** and **(F)**, cells were restimulated with cell stimulation cocktail in the presence of plus protein transport inhibitors and analyzed for the intracellular expression of IFN-γ and IL-17. **(G)** and **(H)** CellTrace Violet labeled CD4^+^ or CD8^+^ T cells were stimulated with anti-CD3 and IL-2 in the presence of DMSO or increasing dose of Ifidancitinib for 4 (d) Proliferation of the T cells were measured by dilution of CellTrace Violet. **(I)** and **(J)** CellTrace Violet dye stained CD45.1 T cell blasts from B6 Cd45.1 were adoptively transferred to B6 CD45.2 recipients. CD45.2 recipients were then treated with Ifidancitinib and 20µg rhIL-2. Proliferation of the CD45.1 T cells were measured by dilution of CellTrace Violet. **P < 0.01, ***P < 0.001 (one-way ANOVA). The results are representative of two separate experiments.

Ifidancitinib is a selective next-generation JAK1/3 inhibitor predicted to disrupt γc cytokine and IFN-γ signaling. We characterized the effects of selective inhibition of JAK/STAT signaling in mouse T cells by treatment with Ifidancitinib. We found that treatment of mouse T cells with Ifidancitinib over a range of doses strongly inhibited IL-2-stimulated STAT5 phosphorylation (**Figure 1A**). Ifidancitinib also exhibited potent inhibitory effects on JAK1/2-mediated IFN-γ signaling, as shown by its suppression of STAT1 phosphorylation upon stimulation with IFN-γ (**Figure 1B**). These findings demonstrate that Ifidancitinib is a potent JAK1/3 inhibitor.

To directly test the effect of Ifidancitinib-mediated JAK1/3 inhibition on the development of NKG2D^+^CD8^+^ T cells *in vitro*, we treated IL-15 stimulated T cells with Ifidancitinib at the start of culture. The presence of NKG2D^+^CD8^+^ T cells was quantified after 4 days, and as expected, IL-15 robustly induced the generation of NKG2D^+^CD8^+^ T cells (**Figure 1C**). Compared to vehicle control, however, Ifidancitinib treatment significantly reduced the population of NKG2D^+^CD8^+^ T cells in a dose-dependent manner (**Figure 1D**). Accordingly, the proportions of IFN-γ producing CD8^+^ T cells were also markedly reduced by Ifidancitinib treatment (**Figures 1E, F**). These results indicated that Ifidancitinib-mediated JAK inhibition suppressed the generation and differentiation of IFN-γ producing NKG2D^+^CD8^+^ T cells *in vitro*.

### Ifidancitinib impaired murine T cell proliferation *in vitro* and *in vivo*

C3H/HeJ mice with AA exhibit a striking cutaneous ly0mphadenopathy with increased total cellularity, which may be mediated by a milieu of γc cytokines and other helper cells (7). Therefore, we examined the effect of Ifidancitinib on *in vitro* T cell proliferation driven by IL-2, a known γc cytokine important for T cell proliferation (18). CellTrace Violet-labeled T cells were co-stimulated with anti-CD3 Ab and IL-2 and subsequently treated with either Ifidancitinib or vehicle control. As determined by dye dilution and subsequent flow cytometry analysis, IL-2 strongly promoted T cell proliferation in the vehicle control as expected (**Figures 1G, H**). In contrast, Ifidancitinib strongly suppressed T cell proliferation in a dose-dependent manner (**Figures 1G, H**). Next, to test whether Ifidancitinib could block γc cytokine-driven T cell proliferation *in vivo*, we adoptively transferred CellTrace Violet-labeled B6 CD45.1 T cell blasts into B6 CD45.2 recipient mice, which were then treated with IL-2 for two days in order to promote T cell proliferation (18). Recipient mice were also treated with varying doses of Ifidancitinib over the same time period and the proliferation of transferred T cells in their lymphoid organs was determined after the two days of treatment. As determined by dye dilution, Ifidancitinib inhibited IL-2-driven T cell proliferation in a dose-dependent manner (**Figures 1I, J**). Taken together, these results indicated that Ifidancitinib robustly inhibited IL-2-driven mouse T cell proliferation both *in vitro and in vivo*.

### Ifidancitinib impaired human T cell function *in vitro*

We next evaluated the effects of Ifidancitinib on human T cells. Purified CD8^+^ T cells were stimulated with anti-CD3 Ab in the presence of IL-15 to induce NKG2D expression (7). Compared to the control, Ifidancitinib not only robustly inhibited NKG2D expression (**Figures S1A, B**), but also significantly decreased the proportions of IFN-γ producing CD8^+^ T cells (**Figures S1C, D**). Furthermore, Ifidancitinib reduced the proliferative response of T cells in response to anti-CD3 and IL-2 stimulation in a dose-dependent manner (**Figures S1E, F**). These results demonstrate that pharmacological inhibition of JAK1/3 signaling *via* Ifidancitinib impaired human T cell function *in vitro*.

### Treatment with Ifidancitinib prevented the development of AA

**Figure 2:**
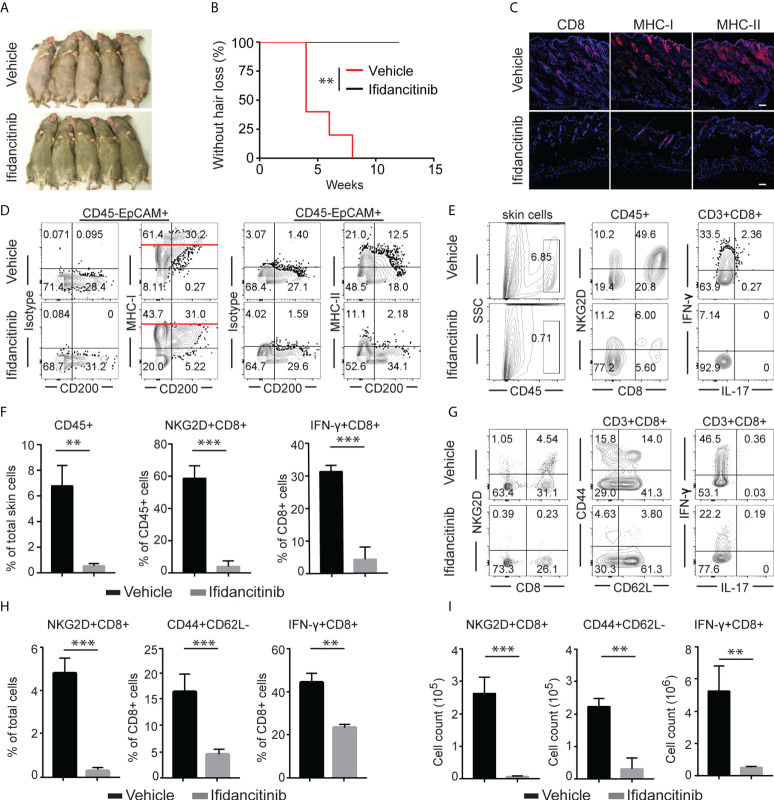
Ifidancitinib prevented the onset of alopecia in AA skin grafted C3H/HeJ mice. Mice were given either Ifidancitinib (n=10) or vehicle control (n=10) in chow diets beginning the day of grafting. AA skin grafted C3H/HeJ mice were given either Ifidancitinib or vehicle control in chow diets for 12 wks. **(A)** The onset of alopecia was inhibited by Ifidancitinib. **P < 0.01, log-rank test. **(B)** Time course of onset of AA in control mice and Ifidancitinib treated mice was shown as weeks after grafting. **(C)** Representative immunofluorescence images of skin sections stained with anti-CD8, anti-MHC-I, or anti-MHC-II mAbs. Dashed scale bars represent 100 µm. **(D)** Representative FACS plots of skin cell suspension showed the expression of MHC class I and II on HF (gated on CD45-EpCAM+CD200+). **(E)** and **(F)** Representative FACS plots of cell suspension of mouse skin and the frequencies of infiltrating CD45^+^ leukocytes and IFN-γ-producing CD8^+^NKG2D^+^ T cell in skin of Ifidancitinib treated mice and control mice. **P < 0.01, ***P < 0.001 (Unpaired Student t test). **(G)** to **(I)** Representative FACS plots of cell suspension of mouse SDLNs **(G)**,the frequencies **(H)** and total cell numbers **(I)** of indicated T cell subsets. **P < 0.01, ***P < 0.001 (Unpaired Student t test).

Given the striking effect of Ifidancitinib on the suppression of γc cytokine-driven T cell proliferation and function, we next tested the effect of Ifidancitinib on disease development in the C3H/HeJ skin graft model of AA. Upon engraftment of AA affected skin from C3H/HeJ AA donor mice, young recipient C3H/HeJ mice were fed either normal chow or Ifidancitinib-incorporated chow diet (0.5 g/kg) (19). In the control group, all mice developed AA by 8 weeks after engraftment. In contrast, none of Ifidancitinib treated-mice developed AA by 12 weeks after engraftment (**Figures 2A, B**). Concordant with the prevention of disease, immunofluorescence staining of skin revealed scant CD8^+^ infiltrates in Ifidancitinib-treated animals, compared to striking infiltration of CD8^+^ T cells in the skin of control mice (**Figure 2C**). Ifidancitinib-treated mice also showed significantly reduced staining for MHC class I and II on HF epithelium compared to controls (**Figure 2C**). Flow cytometry of single cell suspensions of skin samples confirmed reduced expression of MHC class I and II on HF (CD45-EpCAM+CD200+) in Ifidancitinib-treated mice compared to controls (**Figure 2D**) (20). Furthermore, flow cytometry of single cell suspensions of skin samples further showed that CD45^+^ inflammatory infiltrates, including CD4^+^ T cells and CD8^+^ T cells, were significantly diminished in skin from Ifidancitinib-treated mice compared to controls (**Figures 2E, F**). In addition, Ifidancitinib significantly reduced the frequencies and total cell numbers of both CD8+NKG2D+ T cells and CD8+CD44+CD62L- effector memory T cells in skin-draining lymph nodes (SDLN) (**Figures 2G–I**). Taken together, these results indicate that JAK1/3 inhibition by Ifidancitinib prevented disease onset in the C3H/HeJ skin grafted mice.

### Treatment with Ifidancitinib reversed AA in C3H/HeJ mice

**Figure 3:**
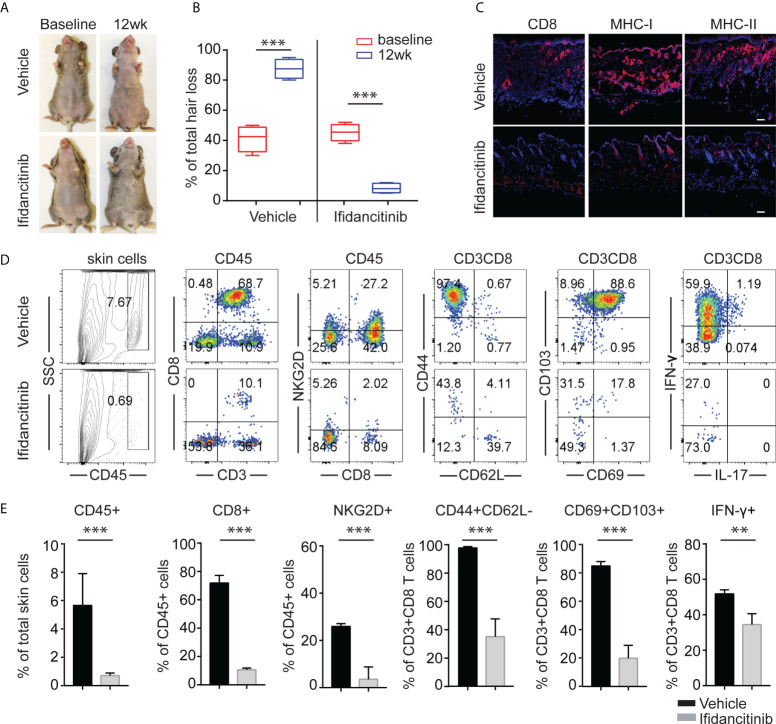
Reversal of established AA with Ifidancitinib treatment. C3H/HeJ mice with AA were given either Ifidancitinib (n=5) or vehicle control (n=5) in chow diets for 12 wks. **(A)** Representative images of Ifidancitinib or control treated C3H/HeJ mice before or after 12 weeks treatment. **(B)** Percentage of skin hair loss or regrowth is shown before and after treatment. ***P < 0.001 (Unpaired Student t test). **(C)** Representative immunofluorescence images of skin sections stained with anti-CD8, anti-MHC-I, or anti-MHC-II mAbs. Dashed scale bars represent 100 µm. **(D)** and **(E)** Representative FACS plots of cell suspension of mouse skin and the frequencies of infiltrating CD45^+^ leukocytes, IFN-γ-producing CD8^+^NKG2D^+^ T cells and T cell differentiation and activation markers in skin of Ifidancitinib treated mice and control mice. **P < 0.01, ***P < 0.001 (Unpaired Student t test).

To address whether Ifidancitinib effectively reverses disease in AA-affected mice, C3H/HeJ mice that developed AA 8-10 weeks after engraftment were treated with Ifidancitinib-incorporated chow diet (0.5 g/kg) (19). Following 12 weeks of treatment, robust hair regrowth was observed in Ifidancitinib-treated mice, compared to sustained and progressive hair loss in control mice (**Figures 3A, B**). Histological analyses on skin biopsies taken after treatment showed that Ifidancitinib-treated mice exhibited substantially reduced AA-associated inflammatory infiltrates and inflammatory markers (CD8, MHC class I and II) (**Figure 3C**). Additional analyses by flow cytometry of single cell suspension of skin samples confirmed that Ifidancitinib significantly reduced inflammatory infiltrates in the skin (**Figures 3D, E**). Role of tissue-resident memory T cells (T_RM_) in pathogenesis of AA was implicated in patients with AA but remains poorly understood (21, 22). The percentage and total number of CD69^+^CD103^+^CD8^+^ T_RM_ were markedly reduced in the skin of Ifidancitinib-treated mice compared to skin of control mice. Flow cytometry showed that the proportions of IFN-γ producing CD8^+^ T cells in the skin were also markedly reduced in Ifidancitinib-treated mice (**Figures 3D, E**).

To gain further insight into the molecular responses to Ifidancitinib, we performed bulk RNA sequencing (RNA-seq) on the skin of treated mice. We found that Ifidancitinib induced dramatic changes in gene expression in treated mice at 10 weeks and shifted gene expression patterns towards the pattern of non-alopecic C3H/HeJ mice (**Figure S2A**). We showed that the sets of genes selected to comprise our IFN and CTL signatures-Cd8a, Gzmb, Icos and Prf1 for the CTL signature and Cxcl9, Cxcl10, Cxcl11, Mx1, and Stat11 for the IFN signature were significantly decreased in mice responsive to treatment with Ifidancitinib (**Figure S2B**) (7). In summary, these results indicate that the JAK1/3 inhibitor Ifidancitinib reduced the presence of inflammatory infiltrates and attenuated their function in skin, leading to effective reversal of established disease.

### Effects of Ifidancitinib on immune cell populations in peripheral lymphoid organs

We next investigated mechanisms underlying the reduction of infiltrating T cells in the skin during Ifidancitinib treatment. We examined whether treatment affected the number, phenotype, or function of T cells or T cell subsets in peripheral lymphoid organs. We found that AA mice treated with Ifidancitinib had a significantly reduced absolute number of total CD3 T cells, CD4 and CD8 T cells in the SDLNs compared with controls (**Figure S3**). CD8^+^NKG2D^+^ T cells and CD8^+^CD44^+^CD62L^-^ effector memory T cells in SDLNs, which are associated with AA pathogenesis, were diminished in terms of both absolute number and percentage of total T cells (**Figure S3**). These results indicate that Ifidancitinib treatment resulted in a profound reversal of T cell activation and proliferation in peripheral lymphoid tissues.

### Treatment with Ifidancitinib induced effector T cell exhaustion

**Figure 4:**
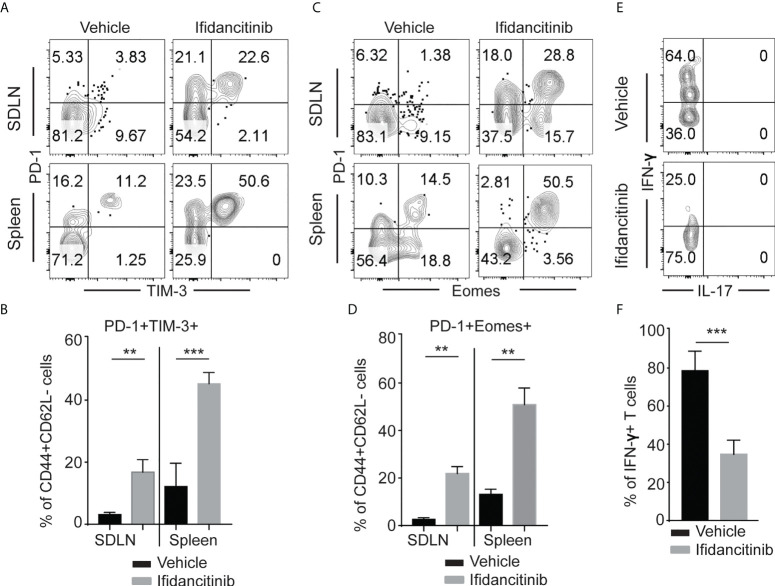
Ifidancitinib induced multiple co-inhibitory receptors in CD44^+^CD62L^-^ CD8^+^ T cells. Mice were treated as in **Figure 3**. **(A)** and **(B)** The frequency of PD-1^+^, Tim-3^+^ and Eomes^+^ T cells within CD44^+^CD62L^-^ CD8^+^ T cells populations within the SDLNs of mice treated with Ifidancitinib or vehicle. **P < 0.01, ***P < 0.001. (Unpaired Student t test). **(C)** and **(D)** The frequency of PD-1^+^, Tim-3^+^ and Eomes^+^ T cells within CD44^+^CD62L^-^CD8^+^ T cells populations within spleen of mice treated with Ifidancitinib or vehicle. **P < 0.01. (Unpaired Student t test). **(E)** and **(F)** The frequency of IFN-γ producing cells within CD44^+^PD-1^+^Tim-3^+^CD8^+^ T cells from Ifidancitinib-treated mice or vehicle treated mice. ***P < 0.001. (Unpaired Student t test).

In the setting of chronic viral infection, which results in the sustained disruption of the γc cytokine network, effector T cells might undergo exhaustion (23). Exhausted T cells display high levels of PD-1 and reduced production of effector cytokines, such as IFN-γ (23, 24). Previous studies showed that Jak3 knockout T cells have impaired γc cytokine signaling and show increased expression of PD-1 and LAG-3 (25). Due to its properties as a potent JAK1/3 inhibitor, we postulated that in addition to inhibiting the generation and proliferation of alopecic T cells, Ifidancitinib might also may also selectively induce effector T cell exhaustion in treated mice. Indeed, we observed an increase in the percentage of CD44^+^ effector T cells coexpressing high levels of PD-1 and TIM-3 in the SDLNs and spleen in Ifidancitinib-treated mice compared to control mice (**Figures 4A, B**). These PD-1-expressing T cells also expressed high levels of Eomes that is known to be highly expressed in exhausted T cells co-expressing inhibitory receptors (**Figures 4C, D**) (26). We next examined cytokine secretion by PD-1^+^TIM-3^+^CD8^+^ T cells in response to stimulation. PD-1^+^TIM-3^+^CD8^+^ T cells from Ifidancitinib-treated mice displayed decreased production of IFN-γ compared to the T cells from control mice (**Figures 4E, F**). Taken together, these findings are consistent with the functional exhaustion of effector CD8^+^ T cells associated with coexpression of PD-1 and TIM-3 and decreased IFN-γ production. Thus, the effect of Ifidancitinib on AA prevention and reversal may be due to the induction of T cell exhaustion, in addition to inhibitory effects on T cell proliferation, differentiation and survival.

### γc cytokines regulated effector T cell exhaustion

**Figure 5:**
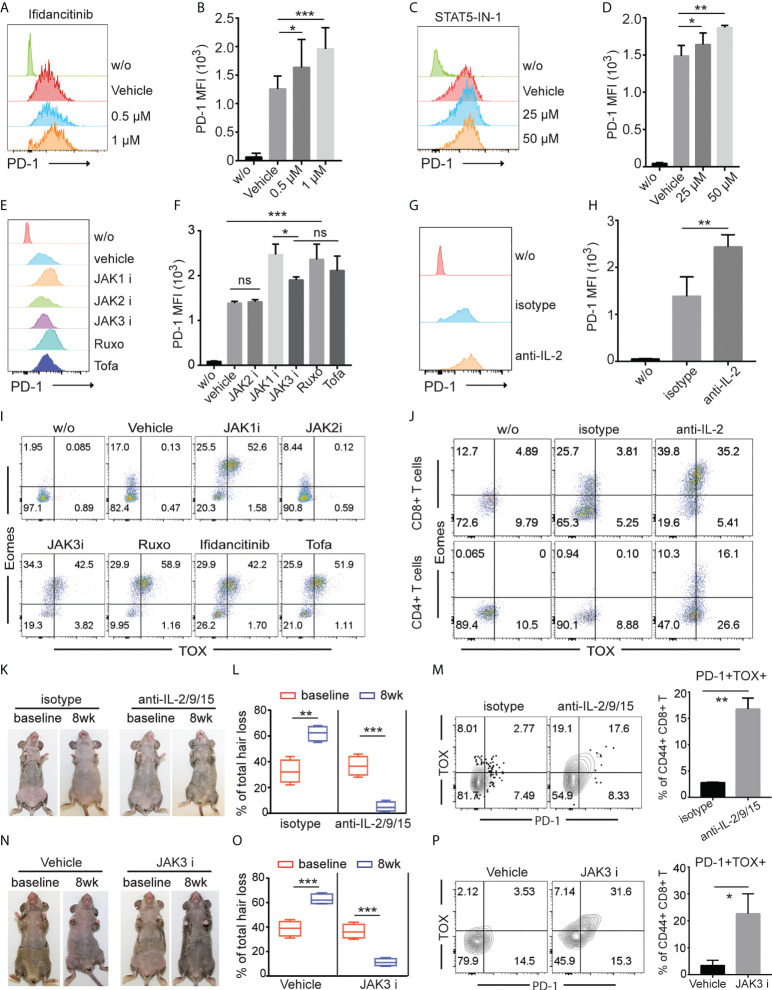
γc cytokines regulated effector T cell exhaustion. **(A)** to **(J)** T cells from C3H/HeJ mice without AA were stimulated with 500 ng/ml anti-CD3 in the presence of indicated regents *in vitro* for 4 (d) **(A)** and **(B)** The expression of PD-1 on CD8^+^ T cells was measured by FACS after treated with increasing dose of Ifidancitinib. *P < 0.05, ***P < 0.001 (one-way ANOVA). **(C)** and **(D)** The expression of PD-1 on CD8^+^ T cells was measured by FACS after treated with increasing dose of STAT5 inhibitor. *P < 0.05, ***P < 0.001 (one-way ANOVA). **(E)** and **(F)** The expression of PD-1 on CD8^+^ T cells was measured by FACS after treated with 1 µM of JAK1i (Itacitinib), JAK2i (Fedratinib), JAK3i (Ritlecitinib), JAK1/2-selective inhibitor Ruxolitinib (Ruxo), or pan-JAK inhibitor Tofacitinib (Tofa). ns indicates not significant, *P < 0.05, ***P < 0.001. P values were determined using one-way ANOVA followed by Brown-Forsythe test. **(G)** and **(H)** The expression of PD-1 on CD8^+^ T cells was measured by FACS after treated with 20 µg/ml IL-2 neutralizing mAbs. **P < 0.01. (Unpaired Student t test). **(I)** The expression of Eomes and TOX in CD8^+^ T cells was measured by FACS after treated with 1µM of JAK1i (Itacitinib), JAK2i (Fedratinib), JAK3i (Ritlecitinib), JAK1/2-selective inhibitor Ruxolitinib (Ruxo), Ifidancitinib, or pan-JAK inhibitor Tofacitinib (Tofa). **(J)** The expression of Eomes and TOX in CD8^+^ T cells was measured by FACS after treated with 20 µg/ml IL-2 neutralizing mAbs and isotype control mAbs. **(K)** to **(M)** C3H/HeJ mice with AA were treated with a combination of IL-2 neutralizing mAbs, IL-9 neutralizing mAbs and IL-15 neutralizing mAbs or isotype for 8 weeks. **(K)** Representative images of anti-IL2/9/15 or isotype treated C3H/HeJ mice before or after 8 weeks treatment. **(L)** Percentage of skin hair loss or regrowth is shown before and after treatment. **P < 0.01, ***P < 0.001 (Unpaired Student t test). The expression of PD-1 on CD8+ T cells was measured by FACS after treated with 20 µg/ml IL-2/9/15 neutralizing mAbs. **P < 0.01. (Unpaired Student t test). The results are representative of two separate experiments. **(N)** to **(P)** C3H/HeJ mice with AA were treated with Ritlecitinib or vehicle systemically for 8 weeks. **(N)** Representative images of Ritlecitinib or vehicle treated C3H/HeJ mice before or after 8 weeks treatment. **(O)** Percentage of skin hair loss or regrowth is shown before and after treatment. ***P < 0.001 (Unpaired Student t test). **(P)** The frequency of PD-1+TOX+CD44+CD8+ T cells within SDLNs were measured with mice that were systemically treated with Ritlecitinib or vehicle for 8 weeks. *P < 0.05. (Unpaired Student t test). The results are representative of two separate experiments. The results are representative of two separate experiments.

To gain insight into the underlying mechanisms of the upregulated co-inhibitory receptor expression on T cells after JAK1/3 inhibition, we stimulated T cells in the presence of Ifidancitinib or vehicle control *in vitro*. T cells increased expression of PD-1 after stimulation. Ifidancitinib treatment increased the expression levels of PD-1 on both CD4 T and CD8 T cells compared to vehicle control (**Figures 5A, B** and **Figure S4A**). Similar to Ifidancitinib, STAT5-IN-1 (STAT5 inhibitor) treatment also increased PD-1 expression on both CD4 T and CD8 T cells (**Figures 5C, D** and **Figure S4B**). Similarly, JAK1 or JAK3 inhibition by JAK1-selective inhibitor Itacitinib, JAK3-selective inhibitor Ritlecitinib, JAK1/2-selective inhibitor Ruxolitinib, or pan-JAK inhibitor Tofacitinib but not JAK2-selective inhibitor Fedratinib (27), increased PD-1 expression on both CD4 T and CD8 T cells (**Figures 5E, F** and **Figure S4C**), indicating that JAK1/3-STAT5 pathway might regulate PD-1 expression on activated T cells (28).

We next evaluated the potential contribution of endogenous γ chain cytokine produced by activated T cells to regulate PD-1 expression on activated T cells. Since activated T cells are the major source of IL-2, we simulated T cells with anti-CD3 and anti-CD28 in the presence of anti-IL-2 neutralizing mAbs. We observed that IL-2 neutralization increased PD-1 expression compare to Abs isotype control (**Figures 5G, H** and **Figure S4D**), indicating that endogenous IL-2 was critical for PD-1 downregulation *in vitro* (28).

Transcription factors thymocyte selection-associated HMG box (TOX) and EOMES have been shown to promote T cell exhaustion in cancer and chronic viral infections (26, 28–30). We measured the expression levels of TOX and EOMES in T cells after individual JAK-selective inhibitor treatment. We observed that JAK1 or JAK3 inhibition by selective inhibitors robustly increased the frequency of Eomes^+^TOX^+^ in both CD4 T and CD8 T cells T cells compared to vehicle and JAK2 inhibitor treatment *in vitro* (**Figure 5I** and **Figure S4E**). Similar to JAK1 or JAK3 inhibition by selective inhibitors, IL-2 neutralization promoted the generation of Eomes^+^TOX^+^ T cells *in vitro* (**Figure 5J**).

To evaluate the potential contribution of γ chain cytokine in regulating PD-1 expression and T cell exhaustion *in vivo*, we treated C3H/HeJ AA mice with a combination of IL-2 neutralizing mAbs, IL-9 neutralizing mAbs and IL-15 neutralizing mAbs (anti-IL-2/9/15). We observed increased frequency of PD-1^+^TOX^+^CD44^+^ CD8 T cells with anti-IL-2/9/15 treated mice compared to isotype control treated mice (**Figures 5K–M**). Further, JAK3-selective inhibitor Ritlecitinib increased frequency of PD-1^+^TOX^+^CD44^+^ CD8 T cells (**Figures 5N–P**). These results indicated that γc cytokine regulates T cell survival and exhaustion, and pharmacological inhibition of JAK1 or 3 signaling or neutralizing γc cytokine promote effector T cell exhaustion (23).

### Single cell RNAseq identifies expansion of exhausted CD8 T cells in skin of mice treated with tofacitinib

**Figure 6:**
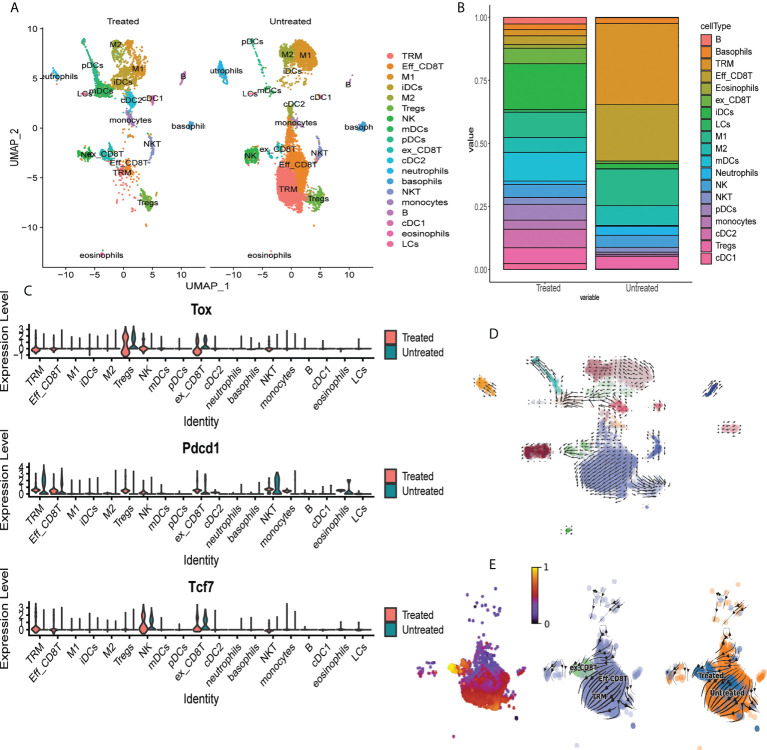
Single cell RNAseq identifies expansion of exhausted CD8 T cells in skin of mice treated with tofacitinib. **(A)** UMAP plots of immune cell clusters identified in the skin of Tofa-treated (Treated) and control mice (Untreated). Note the marked decrease in the proportion of the effector CD8+T cells (Eff_CD8T) and TRM cells, and the expansion of exhausted CD8+ T cells (ex_CD8T) in the skin of Tofa-treated mice. **(B)** Stacked bar plot showing distribution of each cluster relative to the total number of cells per condition. **(C)** Violin plots showing mRNA transcript expression levels of canonical exhaustion markers including *Tox*, *Pdcd1*, and *Tcf7* encoding for TOX, PD1, and TCF1, respectively, across different clusters in Tofa-treated and control mice. RNA velocity analysis reveals that effector CD8+ T cells give rise to TRM and ex_CD8 T cells in AA-affected skin in C3H/HeJ mice. **(D)** RNA velocity plots of immune cell clusters identified in the skin of Tofa-treated (Treated) and control mice (Untreated). **(E)** RNA velocity plots and pseudotime analysis of CD8 T cell clusters identified in the skin of Tofa-treated and control mice.

To determine the effects of JAKi on induction of exhaustion phenotype in CD8+ T cells, we utilized scRNAseq to characterize T cell composition in AA skin after treatment with the pan-JAK inhibitor, Tofactinib (Tofa) (**Figure S5**). We analyzed single cell suspensions of pre-sorted CD45+ cells isolated from whole skin from C3H/HeJ untreated mice or mice treated with Tofa. 19 clusters representing diverse immune cell types have been identified by scRNAseq (**Figure 6A**, **Supplementary Table 1**) (31). We identified a significant reduction in the proportion of TRM cells and Eff_CD8T cells (cluster 0 and cluster 1, respectively) in the skin of Tofa-treated mice, and a significant expansion in the proportion of ex_CD8T cells characterized by the expression of Tox (cluster 9) (**Figures 6A–C**). We used RNA velocity trajectory analysis to predict the origin trajectory of the ex_CD8T cell cluster (**Figures 6D, E**) (32). We identified that similar to TRM cells, ex_CD8T cells in the skin originate from the Eff_CD8T cell cluster (**Figures 6D, E**).

## Discussion

The JAK/STAT pathway plays a critical role in regulating the immune system. As a result, targeting JAK/STAT pathways has shown great promise in the treatment of various autoimmune disorders, including AA (8, 9). We previously found that the lesional skin of AA-affected animals and humans display prominently activated IFN and γc cytokine pathways, both of which are mediated through JAKs (7). This invited trials to target the JAK/STAT pathway for AA treatment, through which we identified selective small molecule JAK inhibitors as a new class of drugs that showed remarkable efficacy in the treatment of AA (7, 12–14).

JAK-mediated γc cytokine signaling is a crucial regulator of lymphocyte development, homeostasis, and function. Specifically, γc cytokines bind to receptors with a unique γc cytokine receptor subunit; the common γc portion of these receptors associate with JAK3 in order to activate STATs for downstream signaling (16). Thus, JAK3 emerged as a potential therapeutic target (33–35), however, recent studies also implicated a role for JAK1 in the pathogenesis of inflammatory diseases (36). JAK1 functions in concert with JAK3 to mediate downstream γc signaling; furthermore, a number of key inflammatory cytokines, such as type I and type II IFN, might depend on JAK1-mediated signaling in several autoimmune disorders, including AA (34). As a result, dual JAK1/3 inhibition would target both the JAK1-dependent inflammatory cytokines as well as the JAK1/3-dependentiactivation of γc cytokine receptor signaling, while sparing JAK2 inhibition (8, 9).

Here, we demonstrated that the JAK1/3 inhibitor Ifidancitinib has potent anti-inflammatory effects both *in vitro* and in C3H/HeJ mice *in vivo*. We showed that JAK1 inhibition by Ifidancitinib suppressed IFN-γ signaling, demonstrated by the reduced expression of IFN-γ-dependent genes such as MHC class I, CXCL10, and CXCL11. IFN-γ is prominently expressed in AA lesions and is believed to contribute to the collapse of HF immune privilege by upregulating the expression of MHC class I in the HF (3, 4, 7). Thus, the preventative and therapeutic effects of Ifidancitinib on AA mice may be in part due to the restoration of HF immune privilege by inhibiting IFN-γ and other forms of JAK1-dependent pro-inflammatory signaling (3, 4, 7).

Furthermore, we found that Ifidancitinib has potent effects on pathogenic T cells that contribute to AA onset and progression. We observed that the *in vitro* differentiation of naïve CD8^+^ T cells to NKG2D^+^CD8^+^ T cells, which are critical to AA pathogenesis (7), was drastically inhibited by Ifidancitinib. We also observed that both the prevention and reversal of AA upon Ifidancitinib treatment were associated with the suppression and reduction of lymphocytes including CD3^+^ T cells, CD4^+^ T cells, CD8^+^ T cells, and NKG2D^+^CD8^+^ T cells as well as CD8^+^CD44^+^CD62L^-^ effector memory T cells that are associated with AA (7). Thus, our results demonstrate that the JAK1/3 pathway is critical to the differentiation of NKG2D^+^CD8^+^ T cells in AA, and that selective inhibition of JAK1/3 is capable of both disease prevention and reversal on the phenotypic level as well as the cellular level.

Recently, efforts to treat inflammatory disease are focused on not only suppressing T cell proliferation and differentiation, but also rendering pathogenic T cells unresponsive to pro-inflammatory signaling (23, 24). One such method is to induce T cell exhaustion, which is characterized by the loss of effector function and the expression of multiple inhibitory receptors such as PD-1, TIM-3, and LAG-3, as well as changes in transcriptional signature (23). Previous studies showed that exhausted, unresponsive CD8^+^ T cells were associated with poor clearance of chronic viral infections as well as cancer cells, but were predictive of good prognosis in several autoimmune diseases (37). The induction of T cell exhaustion remains incompletely understood, however, it has been shown that persistent antigen stimulation, proinflammatory and suppressive cytokines, and regulatory leukocytes all play important roles in driving T cell exhaustion (23). Notably, high expression of PD-1 on HIV-specific exhausted cytotoxic T lymphocytes were associated with the disruption of the γc cytokine network in T cells during HIV infection (38). Since γc signaling is also involved in AA pathogenesis and mediated by the JAK/STAT pathway, we investigated whether Ifidancitinib is able to induce T cell exhaustion. Indeed, JAK1/3 inhibition by Ifidancitinib resulted in increased expression of PD-1 and TIM-3 on CD8^+^CD44^+^CD62L^-^ effector memory T cells. Furthermore, these T cells exhibited reduced IFN-γ production, which is consistent with the loss of effector function observed in exhausted T cells (23, 24). Further, we found that γc cytokines regulated effector T cell exhaustion *in vitro* and *in vivo* through other JAK1 or JAK3 inhibition or by neutralizing γc cytokines. This is consistent with a recent report that STAT5 represses the expression of Tox, Pdcd1, Tim3, Lag3 (39). Reduced common γc cytokine-JAK1/3-STAT5 signaling achieved by JAK inhibitors might be one of the underlying molecular mechanisms that induce T-cell exhaustion.

In AA patients successfully treated with tofacitinib or ruxolitinib, a significant number of patients experience relapse upon discontinuation of treatment (12, 13, 40, 41). While this phenomenon has not been explored in the context of T cell exhaustion, inducing pathogenic T cells unresponsive to pro-inflammatory signaling events that re-establish disease onset may help prevent relapse recurrence (24). Further studies are needed to test the lasting effects of JAK inhibitor induced mediated T cell exhaustion on AA pathogenesis, but the potent loss of effector T cell function induced by Ifidancitinib treatment is encouraging. Treatment regimens that maintain T cell exhaustion after acute treatments may prove to have long-term benefits (23, 24).

In summary, we demonstrate that inhibition of JAK1/3 signaling inhibits the differentiation and proliferation of cytotoxic NKG2D^+^CD8^+^ T cells, induces effector T cell exhaustion, and suppresses immune infiltrates in skin for both disease prevention and reversal.

## Materials and methods

### Mice and study approval

C3H/HeJ, C57BL/6 (B6), CD45.1 (B6.SJL-Ptprca Pepcb/BoyJ) were purchased from The Jackson Laboratory and were maintained under specific pathogen-free conditions at the animal facility at Columbia University Medical Center (CUMC). All protocols were approved by the Institutional Animal Care and Use Committee of CUMC.

### Generation of C3H/HeJ AA mice

Surgical methods for skin grafts to induce AA in C3H/HeJ mice were described previously (7). For the skin graft model, around 10 mm diameter skin from C3H/HeJ AA mouse was grafted to 10-week-old C3H/HeJ recipient and covered with bandage for 10 days. Hair status was examined twice weekly.

### Antibodies and flow cytometry

Antibodies used in the experiments with mouse cells were obtained from Biolegend unless otherwise stated: anti-CD3 (17A2); anti-CD4 (GK1.5); anti-CD8 (53-6.7); anti-B220 (RA3-6B2); anti-CD25 (PC61.5); anti-CD44 (IM7); anti-CD45 (30-F11); anti-CD62L (MEL-14); anti-CD45.1(A20); anti-CD45.2 (104); anti-NKG2D (CX5); anti-PD-1 (29F.1A12); anti-TIM-3 (RMT3-23); anti-CD200 (OX-90); anti-EpCAM (G8.8); anti-MHC I (36-7-5); anti-MHC II (M5/114.15.2); anti-LAG-3 (C9B7W; Thermo Fisher Scientific); anti-pan NK cells (DX5; Thermo fisher); anti-Foxp3 (FJK-16s; Thermofisher); IL-17 (TC11-18H10.1); anti-TNF-α (TN3-19.12); anti-IFN-γ (XMG1.2); anti-Eomes (Dan11mag; Thermo fisher); anti-TOX (REA473; Miltenyi Biotec), anti-pStat1 (Tyr701) (58D6; Cell Signaling); anti-pStat5 (Tyr694) (C71E5; Cell Signaling); anti-Actin (SCBT). The following mAbs raised against human antigens were all purchased from Biolegend: CD3 (OKT3); CD4 (OKT4); CD8 (SK1); NKG2D (1D11); IL-17 (BL168); IFN-γ (B27). Viable cell populations were gated based on forward and side scatters and by fixable blue (Thermo Fisher Scientific) staining. Samples were collected on an LSR II and analyzed with FlowJo software (Tree Star).

### Cytokines and JAK inhibitor

Recombinant murine and human IL-2, IL-15 and IFN-γ were from Peprotech. Ifidancitinib chow diet and Ifidancitinib were provided by Rigel Pharmaceuticals and Aclaris Therapeutics. Itacitinib (catalog HY-16997, MedChemExpress), Fedratinib (catalog 202893, Medkoo), Ritlecitinib (catalog PZ0316, MilliporeSigma), Ruxolitinib (catalog S1378, Selleck), Tofacitinib (catalog 200811, Medkoo)

### Mice treatment

Newly grafted C3H/HeJ mice or long standing C3H/HeJ AA mice were fed with Ifidancitinib incorporated chow diet (0.5 g/kg) for indicated time. Mice were scored weekly for signs of hair regrowth and loss. Mice were euthanized and organs were collected for analysis after treatment.

Ritlecitinib was delivered through an ALZET osmotic pump (MODEL 1002, DURECT). For topical treatment, C3H/HeJ AA mice were topically treated with 2% (w/w) Tofacitinib in Aquaphor (Aquaphor) twice daily. For antibody treatment, newly onset C3H/HeJ mice with AA were administered through IP injection with 0.1 mg combination of IL-2 neutralizing mAbs (JES6-1A12; Bioxcell), IL-9 neutralizing mAbs (9C1; Bioxcell) IL-15 neutralizing mAbs (AIO.3; Bioxcell) or Rat IgG2a (Bioxcell) twice weekly for 8 weeks.

### Mouse tissue processing

Skin was finely minced and digested for 45 min at 37°C with 2 mg/ml collagenase type IV (Worthington) and 0.05 mg/ml DNase I (Sigma-Aldrich) in RPMI 1640. Digested skins were filtered through a 70 μm cell strainer. SDLNs or spleens were homogenized and filtered through a 70 μm cell strainer. Splenocytes were depleted of erythrocytes by RBC Lysis Buffer (Thermo Fisher Scientific). Cells were either re-suspended in FACS buffer (PBS with 2% FCS) for flow cytometric staining, stimulated with PMA and ionomycin for cytokine production or single-cell library construction.

### *In vitro* mouse T cell stimulation, differentiation and cell proliferation assay *in vitro*

For NKG2D^+^CD8^+^ T cell differentiation, purified C3H/HeJ naïve CD8^+^ T cells were stimulated with IL-15 (20 ng/ml) in the presence of Ifidancitinib or vehicle control (DMSO) for 4 d. For *in vitro* proliferation, CellTrace Violet-labeled T cells were stimulated with plate-bound anti-CD3 Ab (0.5 μg/ml) and IL-2 (5 ng/ml), and cell proliferation was assessed by dye dilution. For PD-1 induction, purified C3H/HeJ naïve CD4^+^ T or CD8^+^ T cells were stimulated with 500 ng/ml anti-CD3 in the presence of indicated regents *in vitro* for 4 d described in **Figure 5**.

### Adoptive transfer of T cell subsets and *in vivo* proliferation

Purified T cells from CD45.1-congenic B6 mice were stimulated with plate-bound anti-CD3 Ab (1 μg/ml) and IL-2 (5 ng/ml) *in vitro* for 3d. CellTrace Violet-labeled T cells were adoptively transferred i.v. to B6 (CD45.2) mice. After 12 h, mice were treated with various dosages of Ifidancitinib and 20 μg rhIL-2 as described previously. On day 3, mice were sacrificed and the proliferation of donor T cells from their spleens and SDLNs were analyzed by flow cytometry.

### Intracellular stimulation

SDLN or skin single cell suspensions were cultured with 1× Cell Stimulation Cocktail (Thermo Fisher Scientific). After 1 h, 1× Protein Transport Inhibitors) (500X) (Thermo Fisher Scientific) was added, followed by additional 4 h incubation at 37° C. The cells were then fixed, and permeabilized using FoxP3 fixation/permeabilization kit (Thermo Fisher Scientific) and stained intracellularly with anti-IFN-γ, IL-4, IL-17 or TNF-α for 60 min at 4° C.

### Stimulation of human T cells

Purified human CD8^+^ T cells were stimulated *in vitro* with IL-15 (20 ng/ml) in the presence of Ifidancitinib or vehicle control (DMSO) for 4 d. The frequency of NKG2D^+^CD8^+^ T cell was assessed by flow cytometric analysis. For cell proliferation, CellTrace Violet-labeled T cells were stimulated with plate-bound anti-CD3 Ab (1 μg/ml) and IL-2 (10 ng/ml), and cell proliferation was assessed by dye dilution.

### Immunofluorescence staining and western blot

Immunofluorescence staining of frozen skin sections was performed as previously described., The individual primary Ab was used at optimal concentrations for detection (final concentration 10ug/ml), followed fluorochrome-conjugated goat anti-rat secondary Abs (Thermo Fisher Scientific). The immunofluorescence staining of mouse MHC class I was performed using biotin labeled mouse anti-H-2Kk, followed by fluorochrome-conjugated streptavidin (Thermo Fisher Scientific). Murine T cell blasts were pretreated in the absence or presence of Ifidancitinib (0.1, 0.3 and 1 μM) for 1 h and then stimulated with IL-2 (20 ug/ml) or IFN-γ (40 ug/ml) for 15 min. Total cell lysate (40 ug) was separated by electrophoresis and probed with Abs as described previously.

### Mouse skin bulk RNA sequencing and bioinformation analysis

Total cellular RNA was extracted using RNeasy Kit (Qiagen) from skin homogenates. RNA quality and quantity were determined using an Agilent BioAnalyzer (Agilent Technologies). RNA isolation, library construction and sequencing were performed at GENEWIZ. Gene-expression analysis further was performed with the Seurat R package (27).

### Single-cell library construction

To prepare mouse skin single-cell suspensions of immune cells, live CD45+ cells were FACS sorted. The samples were submitted to the JP Sulzberger Columbia Genome Center’s Single Cell Analysis Core. There, the Chromium Next GEM Single Cell 5’ Reagent Kit V2 was used to prepare cell libraries according to the manufacturer’s instructions, with a target of 5000 cells and 350 million reads. Libraries were sequenced on an Illumina NextSeq 500/550.

### Bioinformatics analysis

After sequencing, FASTQ files were processed using the CellRanger v6.1.2 and aligned to the mm10-2020-A reference transcriptome. The output CellRanger files of the individual samples were merged into a single Seurat object and analyzed using Seurat package v4.0.6 in R v4.1.0 (31). For quality control, we removed cells with lower than 500 molecules detected within a cell and cells with more than 5% of UMIs were derived from mitochondrial genes, and included cells which had more than 200 genes but less than 3000 genes. Post quality control, 7127 cells for untreated skin and 4230 cells for Tofa-treated skin remained for downstream bioinformatics analyses. We then used Seurat package to integrate the individual samples, perform a global-scaling normalization and linear transformation using ‘NormalizeData’, and ‘ScaleData’ functions, respectively. We next used the ‘Elbowplot’ function, to determine the dimensionality of the dataset, and subsequently used 30 PCAs to cluster the cells using the Louvain algorithm. We then performed uniform manifold approximation and projection (UMAP) dimensionality reduction to explore and visualize the dataset. We used ‘FindAllMarkers’ function in Seurat to find differentially expressed markers for each cluster and used canonical markers to match the unbiased clustering to known cell types as outlined in **Supplementary Table 1**. We used ‘VlnPlot’ and ‘FeaturePlot’ functions in Seurat to show expression probability distributions across clusters and visualize feature expression on a PCA plot.

To perform RNA velocity analysis in our scRNASeq dataset, we used the Python v.3.11 based package scVelo, which uses normalized Seurat object in conjunction with RNA Velocity analysis (32). Briefly, we used Samtools v1.10 and Velocyto to generate Loom files for the individual single cell samples we analyzed using Seurat. Then, we integrated the individual loom files and the Seurat meta-data using ‘anndata’ function. Then, we merged the individual samples into one anndata object and added UMAP coordinates from the merged Seurat object to match the order of the Cell IDs in the anndata object. At last, we used scVelo to generate RNA Velocity plot based on Seurat UMAP coordinates and the ‘subset’ function to generate RNA Velocity plot for the CD8 T cell subsets (cluster 0, 1, and 9).

### Statistical analysis

Statistical analyses were performed using the GraphPad Prism 7.0 software. Data were expressed as mean plus or minus SEM for each group and results were compared with 2-tailed t tests, log-rank test, or one-way ANOVA. n.s.> 0.05, *P < 0.05, **P < 0.01, ***P < 0.001, ****P < 0.0001.

## Data availability statement

The data presented in the study are deposited in the NCBI Sequence Read Archive (SRA), accession number PRJNA863610.

## Ethics statement

The animal study was reviewed and approved by Institutional Animal Care and Use Committee of CUMC.

## Author contributions

ZD and AC conceived the study. ZD, TS, YC, and EL performed the experiments. TS, YC, EL, and EW analyzed data and provided critical review of the manuscript. ZD and AC analyzed data wrote the manuscript. AC supervised the study and provided funding. All authors contributed to the article and approved the submitted version.

## Funding

This work was funded National Institutes of Health grant numbers P50AR070588 Alopecia Areata Center for Research Translation (AACORT) (to AC), as well as Pfizer’s Global Health Grants Pfizer CU21-2940 (to AC) and Pfizer 73035959 (to ZD). The funder was not involved in the study design, collection, analysis, interpretation of data, the writing of this article or the decision to submit it for publication. Additional supports were obtained from Locks of Love. ZD is a recipient of young investigator grant award from National Alopecia Areata Foundation and a K01AR070291 award from NIH/NIAMS.

## Acknowledgments

We thank Emily Chang, Jade Huang and Ming Zhang for expert assistance in the laboratory. We appreciate the support of the Skin Disease Research Center in the Department of Dermatology at Columbia University.

## Conflict of interest

Columbia University has licensed patents on the use of JAK inhibitors in alopecia areata to Aclaris Therapeutics, Inc. AC is a consultant to Almirall, Janssen Arcturus and a shareholder of Aclaris Therapeutics.

The remaining authors declare that the research was conducted in the absence of any commercial or financial relationships that could be construed as a potential conflict of interest.

## Publisher’s note

All claims expressed in this article are solely those of the authors and do not necessarily represent those of their affiliated organizations, or those of the publisher, the editors and the reviewers. Any product that may be evaluated in this article, or claim that may be made by its manufacturer, is not guaranteed or endorsed by the publisher.
