# Supplementary material for: Induction of T cell exhaustion by JAK1/3 inhibition in the treatment of alopecia areata
Source: Front Immunol. 2022 Sep 20;13:955038. doi: 10.3389/fimmu.2022.955038 (PMC9531018; doi:10.3389/fimmu.2022.955038)
Supplement: Supplementary file 1 [file Image_1.pdf]

Supplementary Figure 1

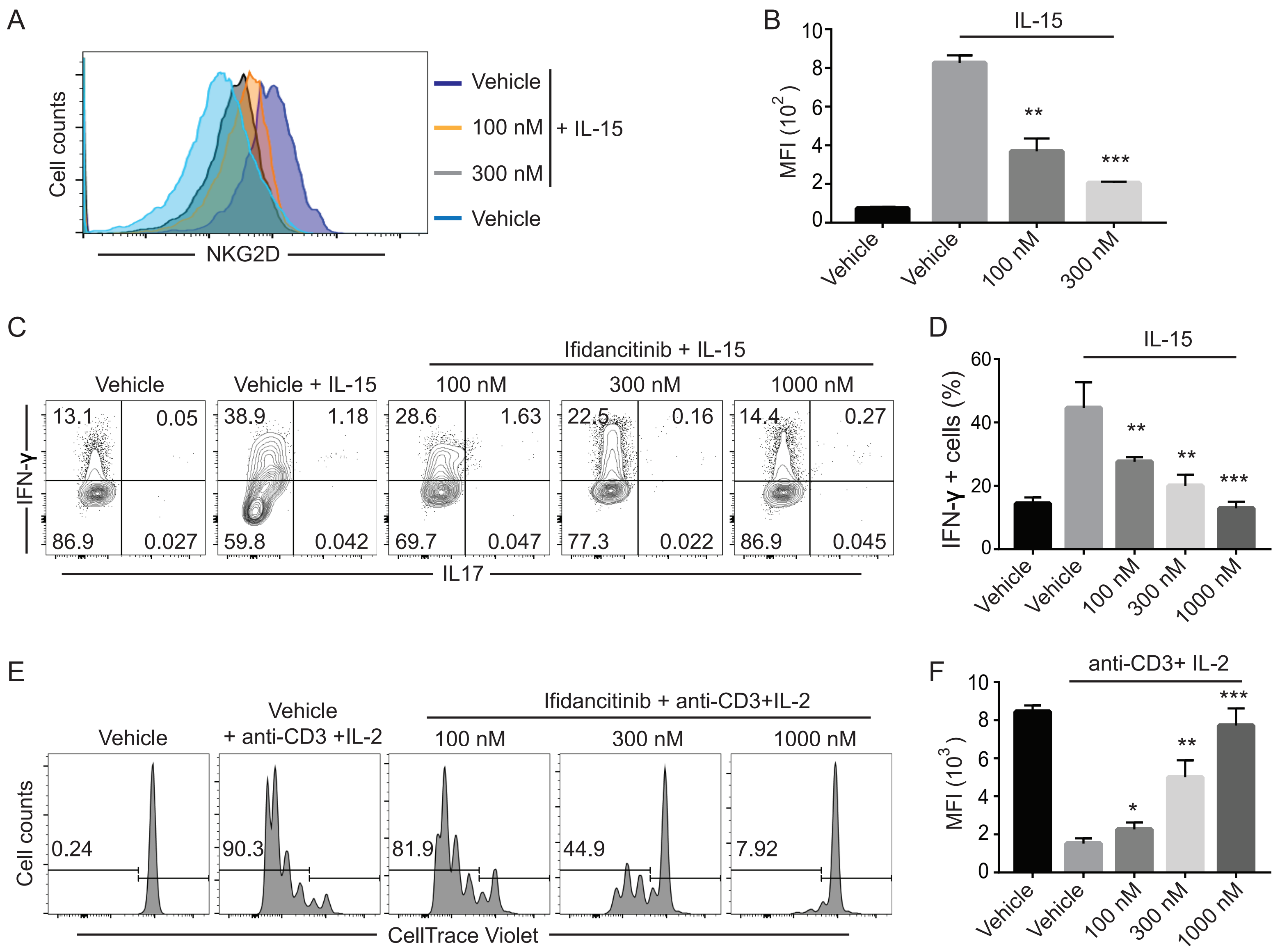

Supplementary Figure 1. Ifidancitinib inhibits human T cell activation and proliferation. CellTrace Violet dye CD8⁺ T cells were stimulated with rhIL-15 in the presence of DMSO or increasing dose of Ifidancitinib for 4 d. **(A)** The NKG2D expression on CD8⁺ T cell was analyzed by flow cytometry. **(B)** The Ifidancitinib or vehicle treated T cells were restimulated with cell stimulation cocktail in the presence of plus protein transport inhibitors and analyzed for the intracellular expression of IFN-γ and IL-17. **(C)** CellTrace Violet labeled human CD8⁺ T cells were stimulated with anti-CD3 and rhIL-2 in the presence of DMSO or increasing dose of Ifidancitinib for 4 d. Proliferation of the T cells were measured by dilution of CellTrace Violet. The results are representative of two separate experiments. *P < 0.05, **P < 0.01, ***P < 0.001 (one-way ANOVA). The results are representative of two separate experiments.
