# Supplementary material for: Induction of T cell exhaustion by JAK1/3 inhibition in the treatment of alopecia areata
Source: Front Immunol. 2022 Sep 20;13:955038. doi: 10.3389/fimmu.2022.955038 (PMC9531018; doi:10.3389/fimmu.2022.955038)
Supplement: Supplementary file 2 [file Image_2.pdf]

Supplementary Figure 2

A

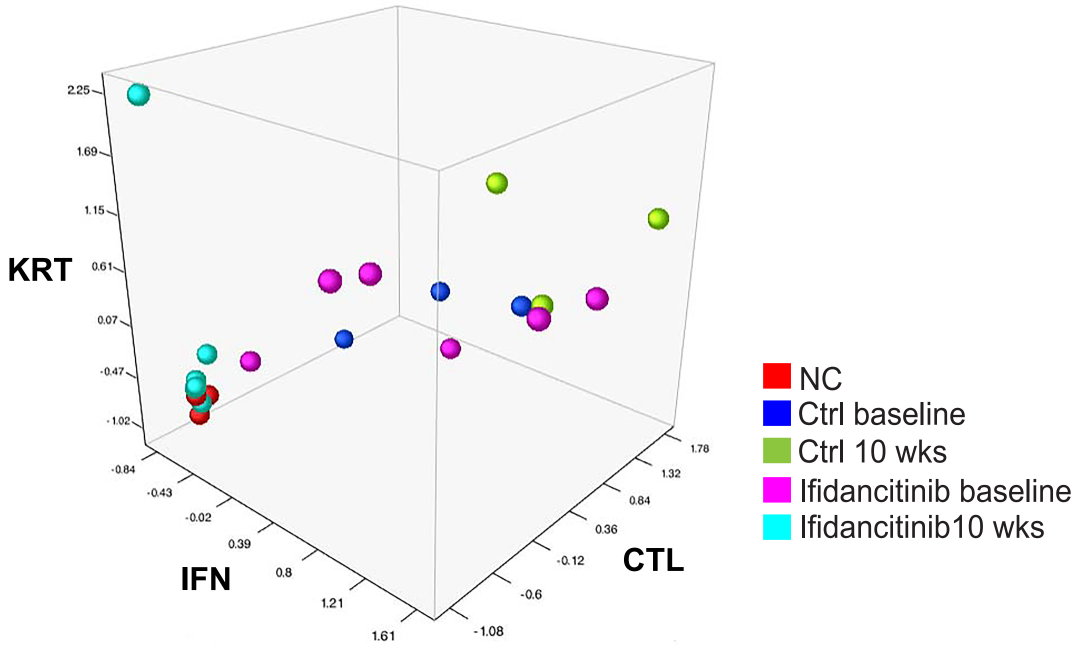

B

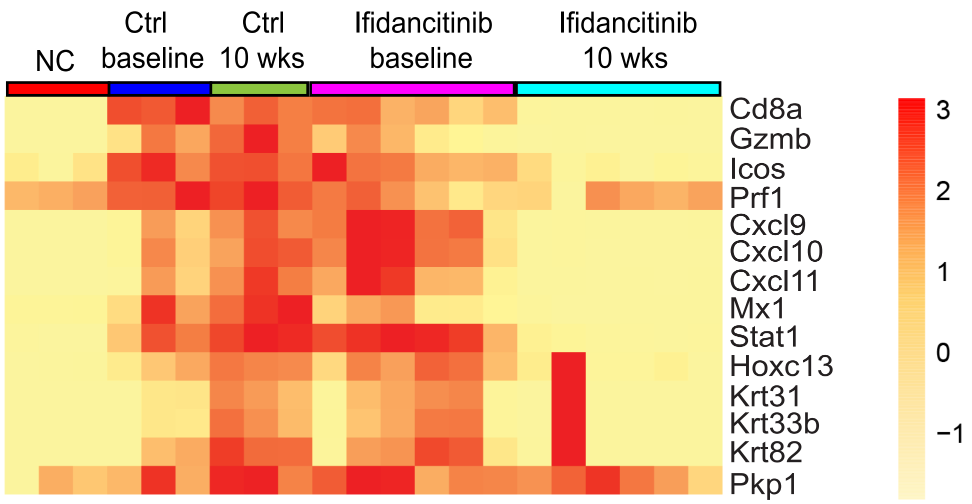

Supplementary Figure 2. Molecular responses to Ifidancitinib. Mice were treated as in **Figure 3**. The bulk RNA-Seq (from mouse whole skin) analysis from treated mice with AA (before treatment versus after treatment versus normal mice) presented as a heatmap **(A)** and as a cumulative ALADIN index **(B)**.
