# Supplementary material for: Induction of T cell exhaustion by JAK1/3 inhibition in the treatment of alopecia areata
Source: Front Immunol. 2022 Sep 20;13:955038. doi: 10.3389/fimmu.2022.955038 (PMC9531018; doi:10.3389/fimmu.2022.955038)
Supplement: Supplementary file 3 [file Image_3.pdf]

Supplementary Figure 3

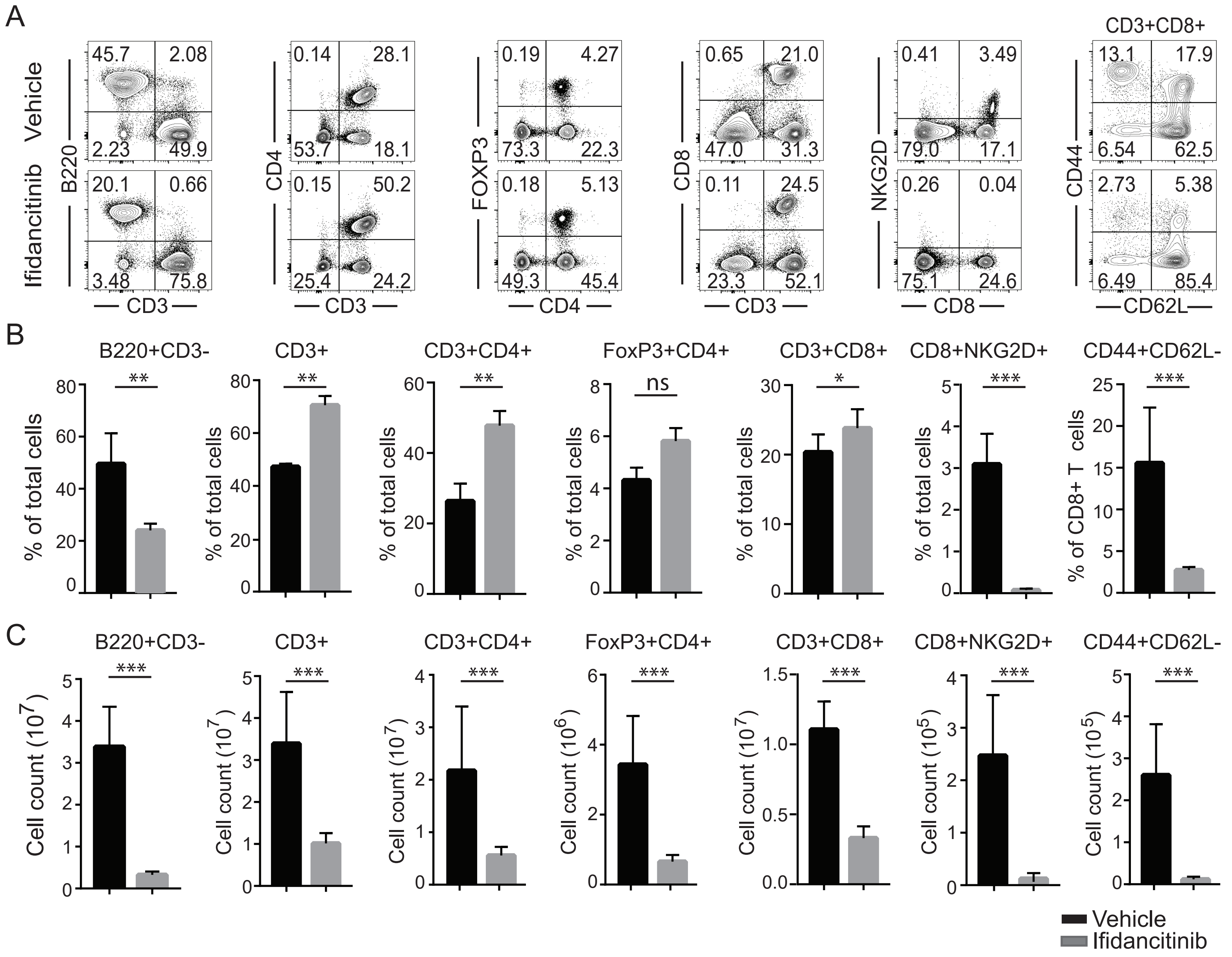

Supplementary Figure 3. Effects of Ifidancitinib on immune cell populations in peripheral lymphoid organs. Mice were treated as in **Figure 3**. **(A)** Representative FACS plots of the SDLNs in the viable cell gate were acquired for each sample. **(B)** The absolute numbers and the percentages of lymphocyte subsets in SDLNs are compared between the Ifidancitinib treated group and control. ns indicates not significant, *P < 0.05, **P < 0.01, ***P < 0.001. (Unpaired Student t test). Data are representative of two experiments with 8 mice total each group.
