# Supplementary material for: Induction of T cell exhaustion by JAK1/3 inhibition in the treatment of alopecia areata
Source: Front Immunol. 2022 Sep 20;13:955038. doi: 10.3389/fimmu.2022.955038 (PMC9531018; doi:10.3389/fimmu.2022.955038)
Supplement: Supplementary file 4 [file Image_4.pdf]

Supplementary Figure 4

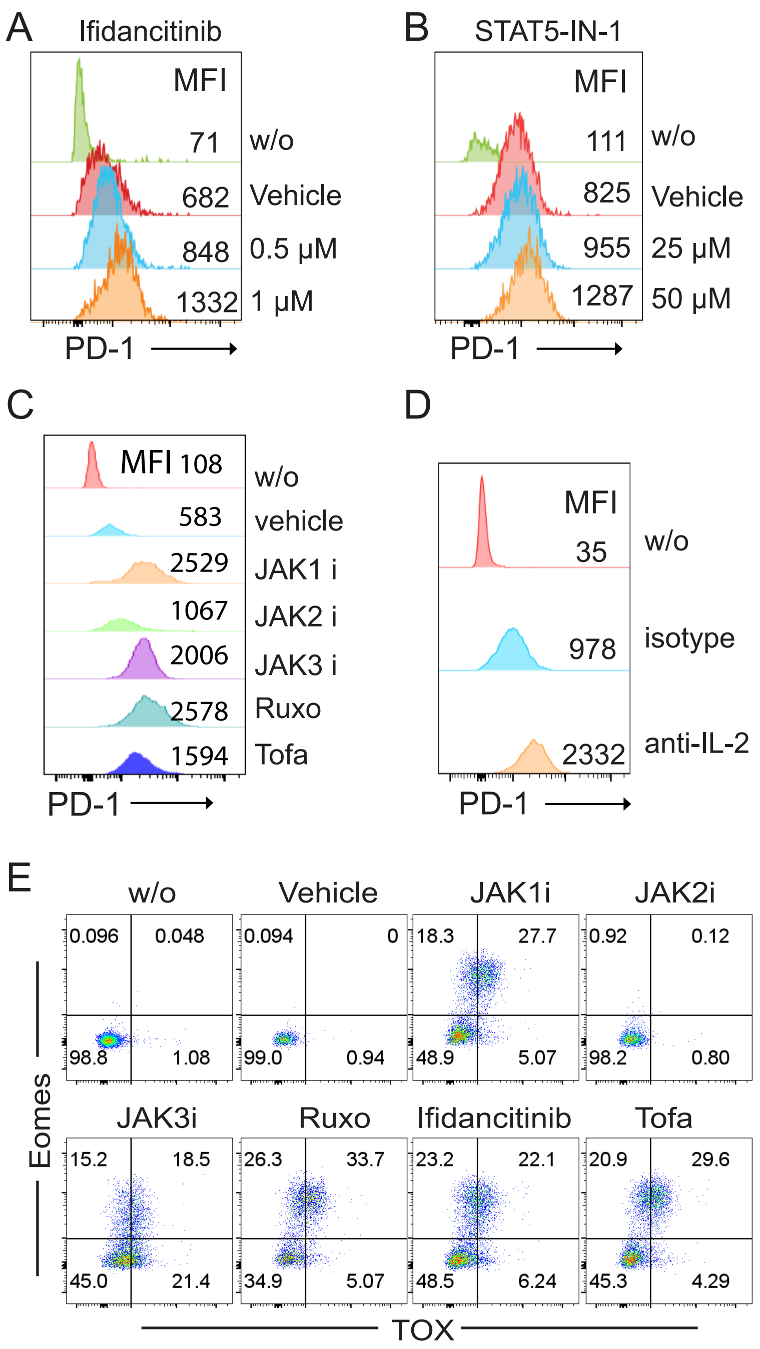

Supplementary Figure 4. γc cytokines regulated effector T cell exhaustion. CD4 T cells were treated as in **Figure**5. **(A)** to **(D)** Representative FACS plots showing the expression of PD-1 on T cells after treated with increasing dose of Ifidancitinib in (A), or treated with increasing dose of STAT5 inhibitor in (B), or treated with 1 μM of individual JAK inhibitors in (C), or treated with 20 μg/ml indicated individual neutralizing mAbs in (D). **(E)** The expression of Eomes and TOX in CD4+ T cells was measured by FACS after treated with 1 μM of JAK1i (Itacitinib), JAK2i (Fedratinib), JAK3i (Ritlecitinib), JAK1/2-selective inhibitor Ruxolitinib (Ruxo), Ifidancitinib, or pan-JAK inhibitor Tofacitinib (Tofa).
