# Supplementary material for: Induction of T cell exhaustion by JAK1/3 inhibition in the treatment of alopecia areata
Source: Front Immunol. 2022 Sep 20;13:955038. doi: 10.3389/fimmu.2022.955038 (PMC9531018; doi:10.3389/fimmu.2022.955038)
Supplement: Supplementary file 5 [file Image_5.pdf]

Supplementary Figure 5

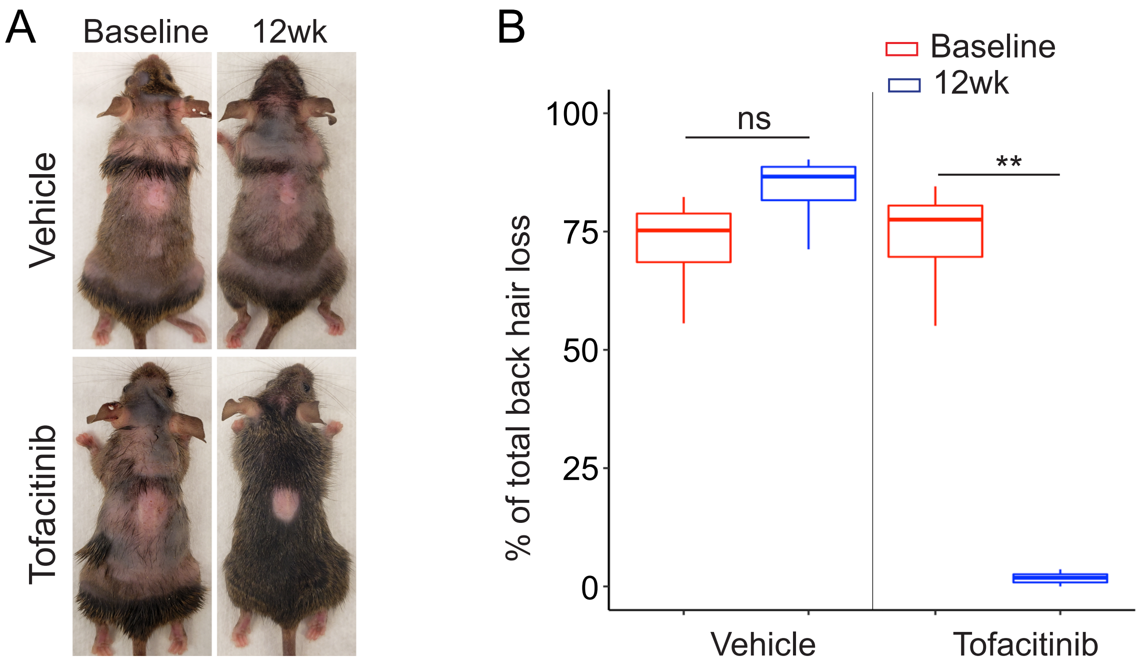

Supplementary Figure 5. Reversal of established AA with topical Tofactinib treatment. C3H/HeJ mice with AA were topically treated with 2% (w/w) Tofacitinib (n=3) or vehicle control (n=3) twice daily for 12 wks. **(A)** Representative images of Tofactinib or control treated C3H/HeJ mice before or after 12 weeks treatment. **(B)** Percentage of skin hair loss or regrowth is shown before and after treatment. ns indicates not significant, $^{**}P < 0.01$ (Unpaired Student t test).
